# Organs-on-a-Chip Module: A Review from the Development and Applications Perspective

**DOI:** 10.3390/mi9100536

**Published:** 2018-10-22

**Authors:** Juan Eduardo Sosa-Hernández, Angel M. Villalba-Rodríguez, Kenya D. Romero-Castillo, Mauricio A. Aguilar-Aguila-Isaías, Isaac E. García-Reyes, Arturo Hernández-Antonio, Ishtiaq Ahmed, Ashutosh Sharma, Roberto Parra-Saldívar, Hafiz M. N. Iqbal

**Affiliations:** 1 Tecnologico de Monterrey, School of Engineering and Sciences, Campus Monterrey, Ave. Eugenio Garza Sada 2501, Monterrey CP 64849, N.L., Mexico; eduardo.sosa@itesm.mx (J.E.S.-H.); angel.vr@itesm.mx (A.M.V.-R.); a00823430@itesm.mx (K.D.R.-C.); a00816656@itesm.mx (M.A.A.-A.-I.); a00824289@itesm.mx (I.E.G.-R.); heran@itesm.mx (A.H.-A.); r.parra@itesm.mx (R.P.-S.); 2 School of Medical Science, Understanding Chronic Conditions Program, Menzies Health Institute Queensland, Griffith University (Gold Coast Campus), Parklands Drive, Southport, QLD 4222, Australia; i.ahmed@griffith.edu.au; 3 Tecnologico de Monterrey, School of Engineering and Sciences, Campus Queretaro, Epigmenio Gonzalez 500, Queretaro CP 76130, Mexico; asharma@itesm.mx

**Keywords:** organ-on-a-chip, biosensors, biomedical, microfluidics, in vivo models, applications

## Abstract

In recent years, ever-increasing scientific knowledge and modern high-tech advancements in micro- and nano-scales fabrication technologies have impacted significantly on various scientific fields. A micro-level approach so-called “microfluidic technology” has rapidly evolved as a powerful tool for numerous applications with special reference to bioengineering and biomedical engineering research. Therefore, a transformative effect has been felt, for instance, in biological sample handling, analyte sensing cell-based assay, tissue engineering, molecular diagnostics, and drug screening, etc. Besides such huge multi-functional potentialities, microfluidic technology also offers the opportunity to mimic different organs to address the complexity of animal-based testing models effectively. The combination of fluid physics along with three-dimensional (3-D) cell compartmentalization has sustained popularity as organ-on-a-chip. In this context, simple humanoid model systems which are important for a wide range of research fields rely on the development of a microfluidic system. The basic idea is to provide an artificial testing subject that resembles the human body in every aspect. For instance, drug testing in the pharma industry is crucial to assure proper function. Development of microfluidic-based technology bridges the gap between in vitro and in vivo models offering new approaches to research in medicine, biology, and pharmacology, among others. This is also because microfluidic-based 3-D niche has enormous potential to accommodate cells/tissues to create a physiologically relevant environment, thus, bridge/fill in the gap between extensively studied animal models and human-based clinical trials. This review highlights principles, fabrication techniques, and recent progress of organs-on-chip research. Herein, we also point out some opportunities for microfluidic technology in the future research which is still infancy to accurately design, address and mimic the in vivo niche.

## 1. Introduction

**Figure 1:**
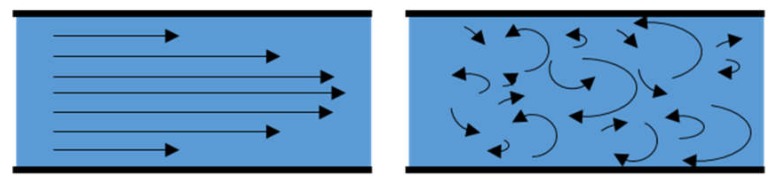
Schematic representation of flow lines in microscale and macroscale. Flow lines follow straight paths in microscale with a parabolic profile; contrary to macroscale, flow lines follow crossing paths with no defined pattern.

In 1990, Manz et al. [1] coined the term “miniaturized total chemical analysis systems (µTAS)” for performing small-volume related reactions. Later, µTAS also encompassed other areas of biology and chemistry. With ever-increasing scientific knowledge and technology advancement, a broader term—so-called “microfluidics”—came into existence and is now often used in addition to µTAS [2]. Microfluidics has been defined as a science and technology which deals with the behavior, precise control and manipulation of fluids through micro-channels. Generally, the fluids are geometrically constrained to a small (10^−9^ to 10^−18^ litters) amount using channels with tens to hundreds of micrometers in dimensions [3]. In microfluidics, the “Lab-on-a-Chip” is an adaptation from microchips to miniaturize laboratory experiments. Following that, other applications appear, and new organs-on-a-chip concept has become very popular among researchers around the globe. This concept developed quickly because its applications to biology and medicine provide tools that are portable, cost-effective, reduce time and can also provide better mimicking of environment for cells. In general, organs-on-a-chip allows the performance of in vitro experiments with more controlled parameters. Although the domain of the device, other equipment is required, such as pumps, incubators, microscopes, and specific target experiment tools. The handling equipment needed for microfluidic devices make it a laboratory tool that cannot leave laboratory facilities. The significant advances in microfluidics began with research about physics and involved handling very low sample volumes, typically a volume range from microliters to femtoliters. For example, the comparison of bulk flow at microscale is remarkable. For instance, turbulent flow only exists in macroscales, and laminar flow is dominant in microscale (see Figure 1), subject to the Reynolds number. The Reynolds number is a critical dimensionless quantity in fluid mechanics used to predict flow patterns in different fluid flow situations as shown in Figure 1. Reynolds number is the ratio of internal force to viscous force. The internal forces tend to move the particles away from the layer, whereas, viscous forces tend to keep the layers moving smoothly one over the other. At low Reynolds numbers, i.e., smaller than 500, viscous forces dominate, and the flow tends to be laminar (sheet-like) [4]. A series of parallel layers can represent laminar flows without any mixing between them. While, at high Reynolds numbers, i.e., larger than 2000, turbulent forces dominate that results in differences in the fluid’s speed and direction, thus, the flow is fully turbulent [4]. Under these conditions, there are lateral and vertical exchanges between the liquid veins which may sometimes intersect or even move counter to the overall direction of the flow (eddy currents). 

Laminar flow, diffusion, fluidic resistance, surface area to volume ratio and surface tension are among the main physical parameters. These parameters change as a consequence of small scales that require a new understanding of the physical and chemical phenomena. Bulk conditions, namely mesoscale and macroscale, are different from microscale, for instance (detailed information on the phenomena related are presented elsewhere [1,2,3]). Herein, we discuss the microfluidics and its application as a versatile tool to construct an organs-on-a-chip module which in turn mimics its counterpart inside the body. The first part of the review focuses on the manufacturing techniques along with the material used to fabricate microfluidic devices/systems. Following that, electrokinetic phenomena with a detailed description of the electrokinetic theory and its role in the applications towards microfluidics are discussed. The last half of the review mainly focuses on different organs-on-a-chip, and describes the most relevant research in the past few years. The last part highlights the current state-of-the-art whole human-on-chip model. Towards the end, we provide concluding remarks and comment on future perspectives.

## 2. Microfluidics Techniques

In the beginning, microfluidics was implemented by industrial processes such as gas chromatography and printing machines to handle small volumes to perform fast analysis with high precision [3]. Later, lithography from microelectronics was adapted to construct devices with medical potentialities using various types of biocompatible materials. Verpoorte and De Rooij [2] reviewed developments that have emerged from the increasing interaction between the microelectromechanical systems (MEMS) and microfluidics worlds. The incorporation of MEMS techniques to fluidic device fabrication using photo-sensible polymers allows producing molds by shading patterns with UV light. This mold is then used to cast the pattern using a biocompatible polymer. The principal characteristics of polydimethylsiloxane (PDMS) are transparency, flexibility, biocompatible, gas permeable, etc. Through the high attention attracted towards microfluidics in the last two decades led to the development of novel fabrication processes and the use of other materials [5,6,7,8,9]. Despite the significant involvement of physics, the integration of other research areas such as chemistry, biology, and medicine to labs-on-a-chip confronted researchers with new problems which made them develop new microfluidic platforms [8]. Replica molding, along with procedures such as micro-contact printing, casting, injection molding, and embossing, encompass the techniques for manipulating elastomeric structures [10,11]. Some of the first approaches have used micro-contact printing, replica molding, micro-transfer molding, micro-molding in the capillary, solvent assisted micro-molding, phase-shifting edge lithography, nano-transfer printing, decal transfer lithography, and nano-skiving. Since most techniques use polymeric or organic matter known by physics as soft matter, all together these techniques are known as soft-lithography [9]. Soft-lithography is a rapid prototyping technique applied to generate micro and nanostructures. Generally, a low-cost polymer is used to build the desired pattern. 

### Advantages and Challenges: Traditional vs. Microfluidic Cell Culture Approach

**Figure 2:**
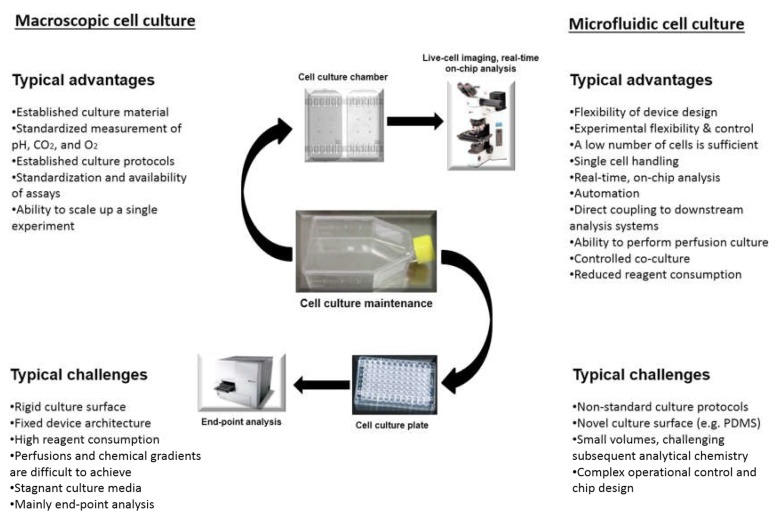
Overview of the advantages and challenges of both macroscopic and microfluidic cell culture. Reproduced from Halldorsson et al. [12], with permission from Elsevier.

Cell culture refers to the cell growth and maintenance of influencing parameters in a controlled environment. In cell related experimental biological research at large, and cell culture technology, in particular, in vitro cell culture models are considered the backbone of the field [12]. Based on literature evidence, several traditional approaches, i.e., (1) culture in the flasks, (2) culture in the dishes and (3) well-plates, etc. have been developed and have gone through heuristic optimization. The modern cell culture techniques such as microfluidics-based cell culture approach offer unique potentialities to culture, maintain, and analyze the cultures in a more sophisticated manner at micro-level. These interactions are not easily replicated or controlled in the traditional cell culture formats [13]. As compared to the traditional cell culture methods, microfluidics-based cell culture approach reveals a clear understanding of an interplay between cell culture parameters and the microenvironmental elements which traditional cell culture methods fails to demonstrate on their own. Furthermore, it is also believed that the controlled operational conditions at microenvironmental level by microfluidic approach will further accelerate and advance the cell culture technology [14]. With a variety of cost and resource benefits such as reduced consumption of reagents, smaller volume, and reduced contamination risk, microfluidics-based cell culture offers a unique platform for efficient high throughput experimentation [13]. The microfluidics-based cell culture approach has several advantages over traditional or macroscopic cell culture. However, both suffer from some considerable challenges. Figure 2 illustrates a comparative overview of significant advantages and challenges of microfluidics-based cell culture approach vs traditional/macroscopic cell culture [12].

## 3. Three-Dimensional (3-D) Printed Microfluidics

Owing to key scientific advancements, three-dimensional (3-D) (bio)-printing technology has drawn significant research interests. As compared to other microfluidic approaches such as soft-lithography (as discussed above), 3-D printing technology has simplified the fabrication process of microfluidic devices to a single step [15]. As mentioned earlier, facile sample preparation and handling, separation of liquids, detection and fluid manipulation, or indeed the device fabrication itself are some of the key functions of microfluidic technology [9,15]. A detailed description of each specific function from fabrication to implementation can be found in a paper by Ahmed et al. [9]. Additionally, 3-D printing technology also has numerous other considerable advantages over conventional fabrication of microfluidic devices. For instance, the capability to incorporate multi-(bio)-materials such as living cells and growth factors, aligned embedding of tissue scaffold with controlled porosity, highly defined device generation with high resolution, defined pore structure and distribution into the device, etc. [15,16]. The historical perspective and potential impact of 3-D printing technology on biotechnology and the chemical sciences, both, have been well covered and reviewed by Gross et al. [16], thus are not the focus of current work. From the industrial and commercial markets perspectives, so far, different 3-D printing techniques have been introduced. For instance, stereolithography (SLA), Digital Micromirror Device-based Projection Printing (DMD-PP), Fused Deposition Method (FDM), Two-photon-polymerisation (2PP), inkjet printing, Electron Beam Melting (EBM) or bio-printers, etc. have been exploited to develop microfluidic devices using photocurable resin, photopolymers, thermoplastic polymers, or materials-based hydrogels as their starting materials [15]. However, among these 3-D printing methods, SLA and extrusion-based system dominate the current market [17]. More detailed information on SLA, DMD-PP. FDM, 2PP, EBM, and bio-printers can be found in a paper by Ho et al. [15]. In summary, 3-D printing technology has an enormous potential to revolutionize the way we print/engineer devices with particular reference to bio-printing, and also perceive the challenges of experimental designs, and fabrication of engineered 3-D biological tissues with particular reference to biological studies where spatial control of samples or cells is critical to integrate into 3-D printed microfluidic devices. 

## 4. Potential Materials and Fabrication Techniques

Among potential materials, PDMS, polymethylmethacrylate (PMMA), polycarbonate (PC), polystyrene (PS), polyvinyl chloride (PVC), polyimide (PI), and the family of cyclic olefin polymers have been widely used in microfluidics [9,18,19,20]. The overall characteristics have made them successful in laboratories around the world. Undoubtedly, the unique characteristics of PDMS allow the generation of the precise micro-channels network to develop a microfluidic device [9]. It is essential to be aware that potential candidate materials (See Table 1) are continuously developed and open new possibilities which need to be explored in microfluidic applications.

From the fabrication viewpoint, several methods have been developed and exploited to construct microfluidic systems. Among them, the (1) hot embossing and (2) injection molding methods are considered two major ones. The first (hot embossing) is very similar to thermal nanoimprint lithography (NIL) and characteristically used to fabricate microfluidic-based products and lab-on-a-chip components. It is a low-cost and flexible fabrication method with unique potentialities to build nanoimprint patterns with high-aspect-ratio microstructures. However, in the injection molding method, the microfluidic device is produced by injecting molten material into a mold where it cools and hardens subject to the mold configuration [56]. A range of materials that include metals, glasses, elastomers, thermoplastic and thermo-setting-based polymers have been used to fabricate micro-channels. Generally, the materials with relatively low viscosity in the solution form are preferred because, during injection, they can lead to good contact with the mold resulting in distinct features [57]. Furthermore, injection-based molding methods have also been used, with an advantage, to construct or fabricate plastic based microfluidic channels using PMMA and PC type materials [58]. However, each fabrication method has specific limitations with regard to the properties of the material. For example, in case of above-mentioned major fabrication methods, i.e., hot embossing and injection molding, the characteristics like materials melting temperature, glass transition temperature, and thermal expansion coefficient are considered most influential for a successful fabrication [57]. These parameters are not only crucial for successful manufacturing but also play a vital role in the sealing process where materials are thermally bonded, e.g., in hot embossing method. 

## 5. Electrokinetic Phenomena: Theory and Microfluidic Applications

**Figure 3:**
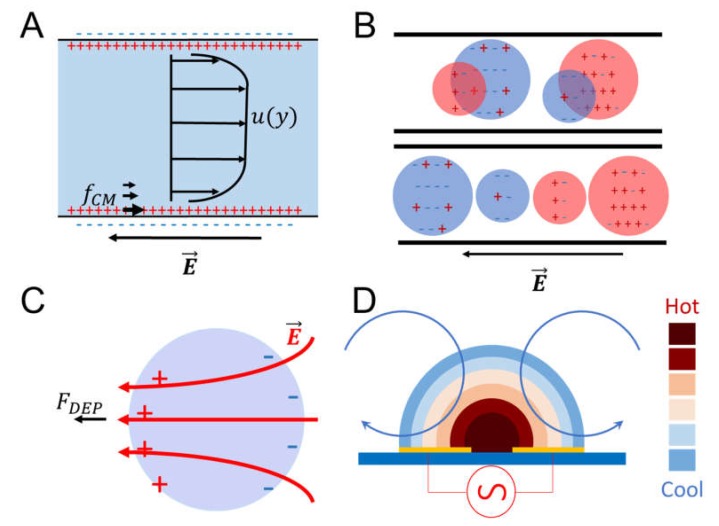
A schematic representation of electrokinetic phenomena. (**A**) electro-osmosis, (**B**) electrophoresis, (**C**) dielectrophoresis, and (**D**) electrothermal in alternate current.

Electrokinetic effects occur in the presence of an interfacial double layer of charges. The phenomena appear when an external force produce a tangential motion of fluid, liquid electrolytes, with respect to an adjacent charged surface. Classically, the electric force used to drive fluid flow, and particle motion [59]. For microfluidic technology, it is fundamental to pump and mix fluids, electrokinetic offers an option to avoid mechanical pumps with electro-osmosis [60]. Additionally, separation of phases or heterogeneous solutions can be done with electrophoresis and dielectrophoresis [61,62]. A principles representation of the main electrokinetic phenomena is presented in Figure 3.

Electro-osmosis (Figure 3A) is used to move and mix fluid charged in electrolytic solutions with specific microfluidic designs as presented in the work of Bazant and Squires. The parallel slip flow $u_{\left|\right|}$ due to the electric field $E_{\left|\right|}$ is given by the Herlmholtz–Smoluchowski equation,

$$u_{\left|\right|}=-\frac{\varepsilon \zeta }{\eta }E_{\left|\right|}$$

In terms of permittivity ε and viscosity η of the fluid, and the zeta potential ζ which can be constant or non-uniform. The result in an aqueous solution gives a speed of 70 µm/s with an electrical field of 100 V/cm and zeta potential of 10 mV [63]. 

For electrophoresis (Figure 3B), the mechanism is the same as electro-osmosis. However, the objective is to move small particles with an electrical charge. As a consequence, different sized and electrical charged particles are displaced at a different speed and can be separated. Lapizco’s group was able to segregate three different microorganisms with microns’ diameter variations in a microfluidic device [64]. Another application in microfluidic is the use of Janus particles of different sizes to mix fluids thanks to the electrophoretic phenomena [65].

Dielectrophoresis (Figure 3C) is a phenomenon driven by polarized particles with or without electrical charge immerse in a non-uniform electric field. The electric field can be applied in alternate current and direct current. This method is widely used for dielectrophoretic analysis of macromolecules, viruses, submicron particulates and recently to separate particles with a different shape, size, and dielectric properties [62]. Polarizability of particles expressed with the Clausius–Mossotti factor estimated with the particle $\varepsilon_{p}^{*}$ and suspension media $\varepsilon_{m}^{*}$ complex permittivity, $\varepsilon^{*}=\varepsilon -\left(j\sigma /\omega \right)$,

$$f_{CM}=\frac{\varepsilon_{p}^{*}-\varepsilon_{m}^{*}}{\varepsilon_{p}^{*}+2\varepsilon_{m}^{*}},$$

where σ and ω are the conductivity and angular frequency of the potential. Dielectrophoresis can be positive or negative and it depends of the coefficient expressed before. In case of low frequency of alternate current and direct current, the factor is approximately to the same equation using switching the complex permittivity with the real conductivities. Force and velocity imposed by the dielectrophoretic effect is described by the equations:

$$F_{DEP}=2\pi \varepsilon_{m}r_{p}^{3}f_{CM}\nabla E^{2},$$

and,

$$v_{DEP}=\mu_{DEP}\nabla E^{2}=\frac{r_{p}^{3}\varepsilon_{m}^{*}}{3\eta }Re\left[f_{CM}\right]\nabla E^{2},$$

where, $\mu_{DEP}$ is the mobility, η is the suspension viscosity and $\nabla E^{2}$ is the electric field gradient used to separate trap and isolate particles [64]. Other electrokinetic phenomena is the electrothermal (Figure 3D), which is a consequence of the Joule heating effect. Electrolytes with conductivities bigger than 0.2 S/m exposed to non-uniform alternate current electric field generates temperature, permittivity and conductivity gradients. The result of the formed gradients induces a force $f_{et}$, this electro thermal force is calculated by the following equation:

$$\langle f_{et}\rangle =-0.5\left[\left(\frac{\nabla \sigma }{\sigma }-\frac{\nabla \epsilon }{\epsilon }\right)\cdot E\frac{\epsilon E}{1+{\left(\omega \tau \right)}^{2}}+0.5{\left|E\right|}^{2}\nabla \epsilon \right],$$

where ω and τ are the frequency and the relaxation time [66].

Electrokinetic phenomena in microfluidics potentize achievement of higher milestones in lab-on-a-chip, point-of-care and organs-on-a-chip. A review focused on mixing through electrokinetics microfluidics summarizes extensive research and designs. The most significant advantages are miniaturized and straightforward design, no vibration and fatigue, integration with other microfluidic devices, low hydrodynamic dispersion, high speed and efficiency [67]. Microvalve is one of the applications of electrokinetics solved by using a Janus particle and electro-osmosis to control the flow in different channels presented in Li’s work [68]. Very recently, Shaegh et al. [69] developed a novel rapid prototyping method to fabricate microfluidic chips from thermoplastic materials with embedded microvalves using laser ablation and thermal fusion bonding. A CO_2_-assisted laser micromachining method was employed to engrave and cut PMMA sheets, together with thermoplastic polyurethane (TPU) film. Aiming to generate a gas-actuated microvalve, the authors have used an unfocused CO_2_ laser beam to fabricate semi-circular fluid channels. Likewise, in another recent study [70], the whole-thermoplastic microfluidic functional elements, including a pneumatic (gas-actuated) normally closed microvalve, a micro-check valve, and a pneumatic dual-phase micropump have been fabricated for lab-on-a-chip applications [70]. Analytical microfluidics in the fields of protein, DNA, bacteria and virus and cells uses electrokinetics to sort, separate, concentrate and fix them for their analysis [71]. More details about alternate current electrokinetics microfluidic designs and theory applied to physiological fluids can be found in Reference [72].

In microfluidics, fluids or fluids-based sample pumping is an essential function for biological fluid handling for lab-on-a-chip and μTAS applications [73,74]. However, subject to a specific application, microscale pumping needs vary over a broader range with certain specifications. For instance, fluids or fluids-based sample pumping from low-power mode to high-power, low-flow-rate to high-flow-rate, and/or low-pressure-flow to high-pressure-flow, etc. To fulfill these demanding requisites for a given application, a variety of fluid driving pumps have been developed and roughly classified into two categories, i.e., (1) mechanical micropumps with moving parts, and (2) non-mechanical micropumps without moving parts [73]. Both categories have been further sub-classified subject to various actuation principles. For instance, the mechanical micropumps can be sub-divided and mainly include (i) piezoelectric, (ii) pneumatic, (iii) thermopneumatic, (iv) electrostatic, (v) electromagnetic and (vi) bimetallic SMA micropumps, among others. Non-mechanical micropumps can be sub-divided and mainly include (i) electrokinetic, (ii) electroosmotic, (iii) magnetohydrodynamic (MHD), (iv) electrochemical, (v) acoustic-wave and surface tension and (vi) capillary micropumps, among others [73,75]. A detailed overview and working mode of the above-categorized micropumps has been comprehensively achieved [73,75], thus these are not the focus of the current research.

## 6. Microfluidic: Lab-on-a-Chip

Use of microfluidics on lab-on-a-chip (LOC) is inherent in the handling of very low volumes. Lab-on-a-chip was used mostly for analysis and biochemical detections, but recently it is aimed at diagnostics. Progress towards a portable diagnostic system is becoming possible as new advances in technologies evolve. As mentioned above, alongside microfluidic systems it is necessary to create integrated pumps, electrodes, valves, electrical fields, and microelectronics. Other examples of applications for lab-on-a-chip are for molecular biology, proteomics, cell biology, among others. A recent study focused on flow cytometry [76] addresses the main problems related to LOC microsystems that require other components to provide independent functionality to achieve point-of-care (POC) chips. They implemented a cytometer that identifies and classifies cells with an electromagnetic field. The chip includes electrodes in channels that separate the cell by dielectrophoresis. However, other components are still needed to achieve the desired goal. Similar to the flow cytometer, a lab-on-a-chip designed to perform a liquid biopsy is presented in Reference [77].

## 7. Microfluidic: Organ-on-a-Chip

**Figure 4:**
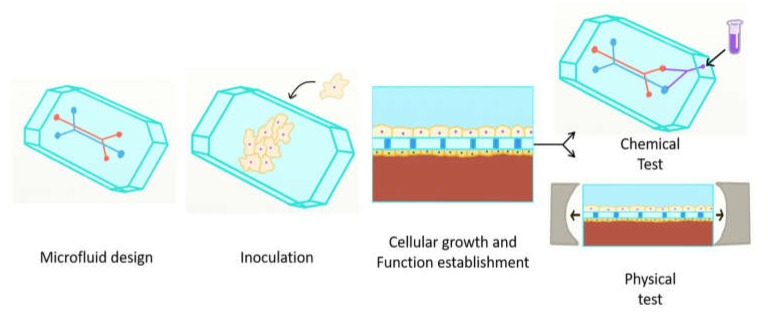
The process to produce different OoCs is in principle the same, taking into account the application. First, the design must address the properties to emulate and measure. Second, different cells must be incubated into the device. Third, cellular growth, differentiation and function are established in order for the chip to operate like an organ. Fourth, data is obtained through chemical and physical testing.

Organs-on-a-chip aim to reproduce the function of biological organs or tissues as realistic models. Cells are grown inside the chambers and channels to generate tissues or complete organs to emulate its biology, and integrative physiology [78]. Achieving a complete functionality involves the inclusion of specific conditions for the organ or tissue such as pressure, flow rate, pH, osmotic pressure, nutrient content, toxins presence, among other properties. Through LOC it has been possible to reproduce several organs-on-a-chip (OoC). Outstanding research to perform in vitro models of cardiovascular, respiratory, nervous, digestive, endocrine and integumentary systems are mentioned in this summary. A general set-up representation of OoC is shown in Figure 4.

### 7.1. Lung-on-a-Chip

Huh et al. [79] constructed a biomimetic microsystem that reconstitutes the critical functional alveolar–capillary interface of the human lung, which is the fundamental functional unit of the living lung. As a proof of principle for a biomimetic microsystems approach, authors have engineered a multifunctional microdevice that reproduces key structural, functional, and mechanical properties of the human alveolar–capillary interface. It was based on a microfluidic system containing two closely apposed micro-channels separated by a thin (10 μm), porous, flexible membrane made of PDMS. The achieved system allowed visualization and characterization of inflammatory processes and response to bacteria [79]. Recently, a bioinspired lung-on-a-chip was capable of reproducing parenchyma by the construction of a thin alveolar barrier in respiratory dynamics. The primary objective to reproduce a lung is to growth epithelial and endothelial cells in cyclic stress that mimics the respiration diaphragm movement of an in vivo model. A bronchial epithelial human cell line from a patient was used, leading to a demonstration of how mechanical stress affects the epithelial barrier permeability. Additionally, there was an improvement of the cell culture in the dynamical model compared to a static one [80]. 

Including a hydrogel micro-layer in the lung-on-a-chip allowed the growth of smooth muscle cells and their interaction with epithelial cells. The combination of type 1 collagen and Matrigel for the hydrogel production was favorable to epithelial cell adhesion. Young’s group was able to incorporate the smooth muscle cells with epithelial cells to evaluate chronic lung diseases [81]. Platforms have been developed to mimic physiological barriers in different systems; a recent review focuses on that matter with microfluidics [82]. Clinical tests for specific diseases can be reproduced with high accuracy in the biochemical reaction chains. A therapeutic model for intravascular thrombosis was set up recently in lung alveolus by Ingber’s group [83]. The model helps to access different drugs; specifically, this group tested antagonist to protease-activated receptor-1 leading to the dissection of complex responses towards antithrombotic drug development.

### 7.2. Liver-on-a-Chip

The liver, with its high metabolic activity, is crucial to life. Its tissue is highly regenerative but suffers major damage from chronic diseases and viral infections. In order to investigate hepatocytes interactions, a microfluidic device was designed to grow hepatic cell cultures in a three-dimensional fashion. This liver-on-a-chip was able to sustain monocultures and co-cultures of hepatocytes and hepatic stellate cells to study its interaction with and without flow [84]. Several other studies are mentioned in the recent review [85] where the liver-on-a-chip applications perform drug analysis, toxicity and screening, pathophysiology and human physiology.

Monitoring metabolic function is an issue to the current in vivo models that only resemble the final result. Therefore, the integration of microfluidics systems like organ and sensors offers an advantage to follow the steps in a biological process. The liver and sensor coupling in the work of Bavli et al. [86] have led to the tracking of the adaptation to mitochondrial dysfunction. The sensor was designed to observe changes in glucose and lactate. Another micro-engineered liver chip was developed to test drug toxicity. The work focused on reconstructing 3-D cellular structure to represent the hepatic sinusoid. The hepatocyte culture was prolonged until 4 weeks and allowed screening cytotoxicity of new drugs [87].

### 7.3. Kidney-on-a-Chip

The kidney is one of the most complex organs to mimic since the integration of several tissues with the correct environmental characteristics is highly difficult. The kidney is formed by glomerular cells, proximal tubule cells, a loop of Henle cells, thick ascending limb cells, distal tubule cells, collecting duct cells, interstitial kidney cells, and renal endothelial cells. However, with four different tissue cells co-cultured in an organization that facilitates their function along with their physicochemical conditions, the kidney can be mimicked. The four tissue types are glomerular, proximal and distal tubule, and collecting duct. One of the most recent studies succeeds in generating a OoC model with glomerular function by using human induced pluripotent stem (hiPS) cells into podocytes and generating glomerular basement-membrane collagen to reproduce tissue–tissue interaction with human glomerular endothelial cells. As a result, they were able to produce an in vitro model to glomerular filtration wall physiology and replicate pharmacologically induced podocyte injury and albuminuria as it occurs in patients [88]. Similar work was done to reproduce the proximal kidney tube for drug and nephrotoxicity probes [89]. Recently, Wilmer et al. [90] reviewed significant aspects of a kidney-on-a-chip development technology for drug-induced nephrotoxicity screening that are crucial for improving the early prediction of drug-induced kidney injury (DIKI). 

### 7.4. Gut-on-a-Chip

Human in vitro models usually fail to represent human gut physiology because of the lack of its natural mechanical microenvironment. The normal gut conditions consist of fluid flow in a complex dynamic caused by its cyclic peristaltic motion. A recent study was able to develop a human gut-on-a-chip with Caco-2 intestinal epithelial cells in flexible microfluidic channels that reproduced the fluidic dynamics and its peristaltic movement. With the described conditions they were able to produce undulating epithelium columns with polarized Caco-2 cells and multiple differentiated intestinal cell types. This work was able to reproduce enteroendocrine cells, Paneth cells, and differentiated Goblet cells that secrete large amounts of mucus found in living small intestine [91,92]. Subsequent work by the same group followed the intestinal inflammation produced by bacterial overgrowth. The project let them to study pathophysiology over a time lapse of weeks, which is an outstanding model for several medical applications [93]. 

Exposure to ionizing gamma radiation through treatment leads to intestinal hemorrhage, sepsis, and death [94]. The radiation of cells from murine models to test damage and measure reactive oxygen species is used to test the properties of the drugs. The gut-on-a-chip application for modeling gamma radiation injury provides an alternative to in vitro testing, and prevents loss of junction continuity and demonstrates similar results [95].

The replication of the coxsackievirus B (CVB1) virus infection is another application of the gut-on-a-chip model. The CVB1 virus causes myocarditis, infects the pancreas and liver, and produces severe problems for neonatal cases [96]. Studies in this field with cells from human lines are a pressing issue, since the use of the human chip to mimic the microenvironment in the dynamic system allows the successfully testing of a polarized infection. The work of Villenave et al. shows how this model can be used for other enterovirus test and the relevance for complex systems to establish better than static cell cultures [97].

### 7.5. Skin-on-a-Chip

The biggest human organ is the skin, which protects the entire body from external conditions. This is one of the most accessible cells for several stress factors causing several reactions. Experiments to test almost every imaginable condition have a great impact on the use of in vitro and in vivo models models, and some of these are not efficient in humans. To reduce animal use and better approach the impact on human skin it is important to provide a better alternative. Skin-on-a-chip is becoming the main in vitro model. An example is a model with epidermal, dermal and endothelial layers developed to reproduce inflammation and edema treated with dexamethasone as a drug testing model [98] (similar work was presented in Reference [99]). The skin is more complex than a general division of epidermis and dermis. Wrinkles are one of the phenomena that happen over time and through external stress. Ultraviolet light, chemicals, physical stimuli, and other processes cause wrinkles. Recent work was able to reproduce wrinkled skin-on-a-chip with the use of magnetic stretching. This work can help to test products for cosmetics and pharmaceutics with a more realistic approximation [100]. The work done by Sriram et al. offers a full-thickness skin chip with novel fibrin-based dermal matrix support for 3-D culture. Additionally, they surpassed problems like inconsistent seeding, epithelial damage, and contraction of the dermal matrix [101].

### 7.6. Brain-on-a-Chip

Human brain function and genetics are very different from animal models. Animal models only provide a basic knowledge of the brain, comparing the human brain/neuronal diseases are not equal, and human models are not an option. A series of research efforts have been directed on producing brain-on-a-chip devices to reproduce fundamental interactions and test applications. Basic research has focused on the principles that command brain formation with high complexity and interconnectivity with other organs. Applications of brain cell function and interaction with drugs for pharmacological proposals to attack degenerative diseases.

The first example is a multilayer device where pluripotent human cells were grown to mimic the central neuron system and incorporate the blood-brain barrier. The complete system was used to analyze the cellular interaction between human fetal neural progenitor cells and the mature model. Then, a chemotactic gradient was included significant for neurodevelopmental studies, neurotoxicology, neuroregeneration, and neuro-oncology research [102]. In the second work, an Alzheimer’s disease in vitro model was reproduced on a chip. The group was able to grow neurospheroids with flow control. Changes in flow led to complex neural network and neural differentiation. Then, amyloid-β toxic effects and treatment were recorded with and without flow. The conclusion was that dynamic conditions provided a better development of the neurospheroids [103]. Similarly, another group was able to generate a multiregional brain-on-a-chip implementing in vivo characteristics. Their chip can develop a particular disease model, evaluating direct electrode signals from specific brain region cells and the network activity [104]. 

### 7.7. Heart-on-a-Chip

Cardiovascular in vitro models usually generate a monolayer tissue culture under static conditions and inside a considerable geometry. With the standard considerations already discussed, the tissue grows with random cell orientation, no flow conditions, and in a flat layer resulting in different physiology than the in vivo conditions. At the beginning of heart-on-a-chip, similar conditions were used but the physiology was improved step-by-step as mentioned in Reference [105]. One of the newest works is a platform where micro-engineered cardiac tissues (μECTs) was created to support a three-dimensional beating tissue from human cardiomyocytes. The mechanical and electrical response showed a high coupling response. During culture, the platform provided mechanical stimuli which resulted in better cell maturation and increased the mechanical and electrical coupling. The device was also used to test several concentrations of isoprenaline [106]. The influence of perfusion condition and microsystem geometry helps to develop cell proliferation with high alignment and morphology [107].

A mussel-inspired 3-D chip was developed thought engineered nanomaterials to test cardiac contractility. The materials used were gelatin as extracellular matrix and titanium oxide and silver nanoparticles. The device can measure in vitro contractile effects by cardiotoxicity of nanoparticles that affect calcium signal to sarcomere [108]. Cardiomyocytes clusters in spheroid geometry are part of an experiment for drug testing in a microfluidic device. The work demonstrates the potential to do studies in the long-term by applying compound concentrations. A 48 hour test was used as a non-invasive assay for quality control in pharmacological applications [109]. In this way, preclinical tests may be used for testing drug efficacy and safety. Kamei et al. built an Integrated Heart/Cancer on a-Chip (iHCC) using human cells. The microfluidic device included micropump and pneumatic valves to reproduce heart dynamics and test the anti-cancer drug doxorubicin (DXR) [110]. 

## 8. Human-on-a-Chip

**Figure 5:**
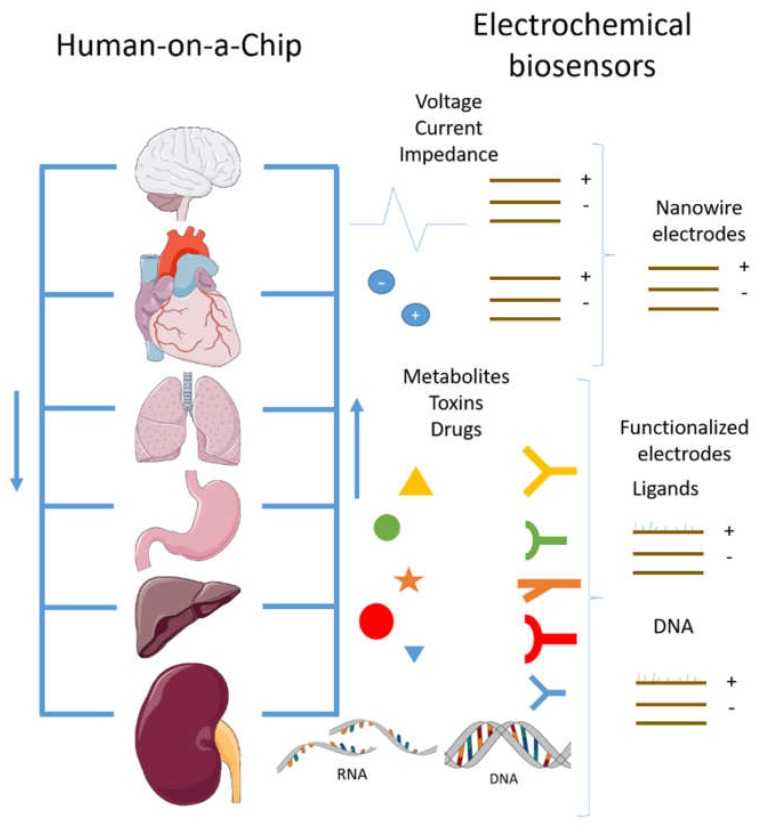
Organs-sensors-on-a-chip microfluidic representation.

Human-on-a-chip refers to an in vitro model of mimicking either normal or pathological whole human physiology within a microfluidic system that has high measurement accessibility and control (Figure 5). Biomedical and pharmaceutical sectors will greatly benefit from multi-organ-on-a-chip and ultimately human-on-a-chip models, in terms of drug development and testing, the reliability of results, overall cost-effective ratio and working efficacy, etc. As discussed earlier, multi-organ-on-a-chip and ultimately human-on-a-chip models are promising alternatives to the animal-based testing model which have been a debating issue for a long time. Another considerable advantage is a low-throughput screening along with a high content screening at a small scale which is often not economical and sustainable on a macroscopic level [111]. Moreover, microfluidics-based chip technology is currently in a mature state and offers exceptional control over culture conditions along with other conditions, i.e., spatial homogeneity, chemical gradients, time-dependent biochemical stimulations, and substrate mechanical properties, etc. [111,112,113,114].

Research is underway around the globe to study the next level of complexity to get insight into the interplay between tissues physically separated in vivo but the circulation-mediated interaction of which could be important for their mutual functionality. Therefore, it is most requisite to construct novel microfluidic designs where tissues must be able to perform the function required to support the other tissues. In order to succeed first, the single organ tissue has been studied separately, and now multi-organ-on-a-chip research has begun with successful results. First by pairs, such as liver-fibroblast, gut-liver, and liver-pancreas, and subsequently by incorporating more organs with an integration factor that includes single and recirculation perfusions, since organ systems communicate by secreting chemical factors and vesicles [115]. Despite the current advancement in technology, some critical challenges for the integration of all the organs in a chip still need to be addressed. For instance, biological challenges include appropriate organ scaling, vascularization of tissues, the inclusion of immune components, the creation of a universal media, induced pluripotent stem cells (iPSCs) sourcing, and consideration of circadian and other cycles on cells, etc. Likewise, technical challenges include connection of platforms to maintain sterility and avoid bubbles, drug adsorption and binding to PDMS, flow rate differences between platforms, and creating ideal oxygenation and nutrient levels for different organs, etc. 

Despite the challenges mentioned above, this growing field of multi-organ-on-a-chip will also help human health care programs around the world [116]. A four-organ-chip system that enables maintain high cell viability and discrete physiological tissue architecture over the entire co-culture period was designed to support absorption, distribution, metabolism, and excretion (ADME). The profiling of substances, along with repeated dose systemic toxicity testing of drug candidates, demonstrates the integrity and functionality of the intestine and the biological barriers of the kidney at a physiologically relevant organ scale. The arrangement enables physiological absorption, first path metabolism in the liver tissue, secondary metabolism and finally excretion through the kidney model and evaluation of pharmacokinetic and pharmacodynamics [117]. A recent review focused on the design parameters to develop a physiologically based pharmacokinetic (PBPK) model used with pharmacodynamics for drug development. This review establishes the basic parametric equations set to represent the human physiology of the human-on-a-chip and considers the critical parameters to be satisfied, along with its limitations [118].

## 9. Concluding Remarks and Future Perspectives

In conclusion, herein we summarize the recent progress in the development of microfluidic-based systems including LOC and multi-organs-on-a-chip. A plethora of microfluidic-based systems has been developed in the past few years with an ultimate aim to facilitate the predictive in vitro and in vivo models. Moreover, comparative to traditional cell culture methods, i.e., (1) culture in flasks, (2) culture in dishes and (3) well-plates, etc. microfluidics-based cell culture approach reveals a clear understanding of an interplay between cell culture parameters and the microenvironmental elements which traditional cell culture methods fail to demonstrate on their own. The versatile multifunctional features of microfluidics—such as precise control over microenvironmental elements—opens up new avenues not only for tissue engineering areas but also for next-generation drug testing sectors. Furthermore, the notable capability of microfluidics to biomimetic the micron-scale structures along with fluidic manipulation under microenvironment demonstrates it to be a powerful tool to engineer products with multifunctional applications. 

One of the most significant targets for research tools development is the human-on-a-chip to replace animal models in research test and pharmaceutical industry. The human-on-a-chip tool offers the opportunities for growth through the incorporation of more tissues with proper function and without external aid. For this proposal, a completely independent system requires that all tissue can adequately provide its physiological function. First, it is needed to sustain cell viability for an extended time. The communication between tissues must offer a similar approach to the in vivo capacity, and not just by the microfluidic channels. For that, a high quantity of cell types must be developed, as shown by some of the presented works, where up to four cell types were grown from stem cells with the correct order and shape. Moreover, a proportional tissue, organ, volume, and whole mass must be sustained to provide a normal scaled human physiology. In our opinion, a new trend would be to incorporate electrochemical biosensors in a human-on-a-chip platform to merge tools (Figure 5). This approach would add a new layer in research studies, allowing innovative experiments on processes such as cancer behavior, congenital diseases, brain function, tissue development, and differentiation. As well as the correct physiology implementations, it is important to consider the design from a physics point of view; for example the inclusion of relevant parameters in the flow system and the biochemical equations. It is necessary to recognize the limitations of these in vitro models through their intrinsic value and exploit their benefits. Current research has been able to get closer to the idea of human-on-a-chip [119], and besides the mainstream of research for drug design and physiology, other novel applications are possible, including sensors development towards the detection of toxins, drugs, and hormones [120]. Through building a human reproductive tract it became possible to demonstrate that the biophysical environment helps sperm to reach the ovule. The cancer model is another critical issue, where an implementation of a human-on-a-chip has let researchers study metastasis, tumor growth, and its physiology. In summary, to fully elucidate and appreciate the potential of human-on-a-chip models as strategic measuring tools to test clinical trials on chips, correlations must be established between human in vitro measurements and traditional in vivo parameters. This will also act as a bridge between conventional cell cultures and new standardized clinical trial procedures without using animal-based models. 

## Figures and Tables

**Table 1:** Potential materials used in microfluidics.

Material	Relevant Property	Proposed Application	Reference
Collagen (Chitosan)	Biocompatibility, versatile control of structure and chemistry	Bio-sensing, film assembly	[21,22]
Silkworm (*Bombyx mori*)	Biocompatibility, mechanically robust, flexibility, high mechanical modulus, and toughness	Fabrication of microfluidic channel	[23,24]
Agarose hydrogel	Lox cytotoxicity, biodegradability, mechanical stability at low solid fractions	Cell culture, sensors, and actuators	[25,26,27]
Teflon	Ease of fabrication with maximum chemical resistance	High precision assay, super clean tools, valves, and pumps fabrication	[28]
Acrylonitrile Butadiene Styrene (ABS)	High resolution, excellent surface finish	Making of the master mold, microfluidics interface (MI), pathogen detection, biological assay	[29,30,31,32,33,34]
Photocurable resin/polymer	Very high resolution with small features	Biology observation of cell growth	[35,36]
ABS, polycarbonate, polyphenylsulfone, elastomers	Cheap material, ease of support removal	Pathogen detection of bacteria and viruses	[37,38]
Polyamide	Fast build speed, multi-material printing, very durable and high-temperature stable material	Making of the master mold	[39,40]
Hydrogels	Swelling and contraction, act as sensors and actuators	Self-regulating valves, microlens arrays, drug release systems, binding of antigens and proteins and glucose. Flow sensors pH regulators, flooding cooling devices.	[29,41,42]
Polyurethane-methacrylate (PUMA)	Economical to manufacture, biocompatible, nontoxic, strong electroosmotic mobility	High-aspect-ratio microstructures	[43]
Polyethylene glycols (PEGs)	Relatively inexpensive, available in a wide variety of molecular weights, biocompatible, negligible cytotoxicity	Microfluidic valves, Channel cover to improve the microfluidic lifetime	[44,45]
Polyhydroxyalkanoates (PHAs)	Biocompatibility, tunable biodegradability	Microfilm barrier for vapor and oxygen	[46]
Gelatin methacrylate (gel-MA)	Photopolymerizable, porous membrane	Mechanistic vascular and valvular biology cell support matrix	[47,48]
Polylactic acid (PLA) and Polyglycolic acid (PGA)	Tunable biodegradation	Porous scaffold for cell culture with better adhesion	[49]
Poly(polyol sebacate) (PPS)	Biocompatibility, design adaptability, mechanical compliance, low cytotoxicity, degradability	3-D microfluidic system, Microbioreactor	[50]
Poly(ethylene glycol) diacrylate (PEGDA) and gelatin methacryloyl (GelMA)	Biocompatibility, neovascularization potential, multi-material fabrication capability at a high spatial resolution	Tissue engineering, regenerative medicine, and bio-sensing	[51]
Poly(methyl methacrylate)	Favorable mechanical and thermal resistance, chemical compatibility	Genomic analysis	[52]
Styrene Ethylene Butylene Styrene (SEBS)	Biocompatibility, Rheological characteristics	Fabrication of complex and more sophisticated microfluidic networks (μFNs)	[53]
Styrene Ethylene Butylene Styrene (SEBS)	Electrical surface properties, stable and relatively high zeta potential magnitude	Microdevices for Electrokinetic Applications	[54]
Styrene Ethylene Butylene Styrene (SEBS)	Reduced drug absorption, Optical transmittance, Mechanical performance	Cell culture	[55]
